# Open reduction and internal fixation of humeral midshaft fractures: anterior versus posterior plate fixation

**DOI:** 10.1186/s12891-019-2888-2

**Published:** 2019-11-10

**Authors:** Sebastian Lotzien, Clemens Hoberg, Valentin Rausch, Thomas Rosteius, Thomas Armin Schildhauer, Jan Gessmann

**Affiliations:** Department of Trauma Surgery and Surgical Research, BG University Hospital Bergmannsheil, Ruhr University Bochum, Bürkle-de-la-Camp-Platz 1, 44789 Bochum, Germany

**Keywords:** Trauma, Humerus, Plate fixation, ORIF, Humeral shaft, Anterior plating, Posterior plating, Radial palsy

## Abstract

**Background:**

Fractures of the humeral shaft represent 2–4% of all fractures. Fractures of the humerus have traditionally been approached posteriorly for open reduction and internal fixation. Reports of treating midshaft fractures with an open anterolateral approach and anterior plating are limited. The purpose of this study was to evaluate a series of humeral shaft fractures treated with plate osteosynthesis regarding the effect of the approach and plate location on the healing rate and occurrence of complications.

**Methods:**

We conducted a retrospective chart review of patients aged over 18 years with humeral midshaft fractures treated with anterior or posterior plate fixation. Selection of the approach to the humerus was based on the particular pattern of injury and soft tissue involvement. The minimum follow-up duration was set at six months. The outcomes included the rate of union, primary nerve palsy recovery, secondary nerve damage, infection and revision surgery.

**Results:**

Between 2006 and 2014, 58 patients (mean age, 59.9; range, 19–97 years) with humeral midshaft fractures were treated with anterior (*n* = 33) or posterior (*n* = 25) plate fixation. After a mean follow-up duration of 34 months, 57 of 58 fractures achieved union after index procedure. Twelve fractures were associated with primary radial nerve palsy. Ten of the twelve patients with primary radial palsy recovered completely within six months after the index surgery. In total, one patient developed secondary palsy after anterior plating, and three patients developed secondary palsy after posterior plating. No significant difference in the healing rate (*p* = 0.4), primary nerve palsy recovery rate (*p* = 0.6) or prevalence of secondary nerve palsy (p = 0.4) was found between the two clinical groups. No cases of infection after plate fixation were documented.

**Conclusions:**

Open reduction and internal fixation using an anterior approach with plate fixation provides a safe alternative to posterior plating in the treatment of humeral shaft fractures. An anterior approach allows supine positioning of the patient and yields union and complication rates comparable to those of a posterior approach with plate fixation for the treatment of humeral shaft fractures.

## Background

**Fig. 1:**
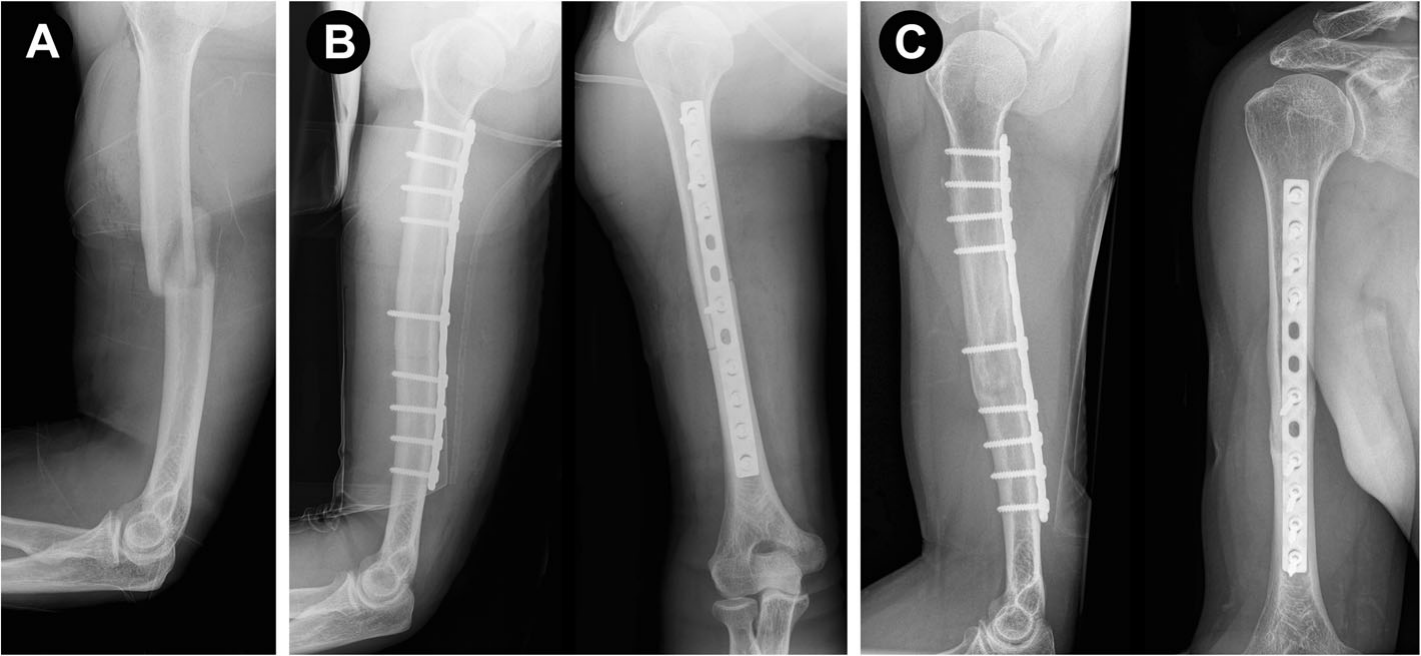
**a**–**c.** A 55-year-old patient with an Orthopaedic Trauma Association (OTA) type 12-A1 humeral fracture (**a**). Postoperative X-rays after open reduction and internal fixation (ORIF) using a limited contact dynamic compression plate (LCDCP) (**b**). Final anteroposterior and lateral views showing secondary fracture healing with callus formation as a result of relative stability of the construct (**c**)

**Fig. 2:**
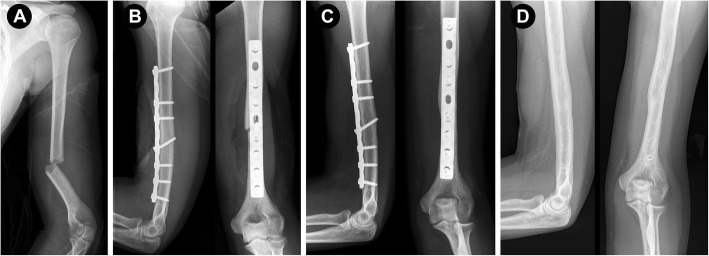
**a**–**d.** An 18-year-old female patient with an OTA type 12-A3 humeral fracture (**a**). Postoperative radiographs after open reduction and internal fixation (ORIF) utilizing an anterior limited contact dynamic compression plate (LCDCP) (**b**). Fracture healing in the same patient six months after ORIF. Although we aimed for absolute stability and primary fracture healing, callus formation, a sign of secondary fracture healing, was observed. **c** Radiographs one year after the index procedure and implant removal (**d**)

Fractures of the humeral shaft represent 2–4% of all fractures [1]. Currently, there are no defined gold standards for the treatment of humeral shaft fractures [2, 3]. While nonoperative treatment has a long and successful history in certain cases [4], new surgical treatment methods have been developed to reduce soft tissue damage, improve early training and prevent long uncomfortable periods of immobilization, which can be associated with nonoperative treatment [3, 5–8]. For open reduction and internal fixation (ORIF), the humerus has traditionally been approached posteriorly. The posterior approach offers biomechanical advantages due to the ability to apply the plate on the tension side of the humerus (Fig. 1a-c) [9]. Nevertheless, there are different surgical approaches for treating humeral fractures [10, 11]. The anterolateral approach [12] and its modifications are widely employed for exposure of the humerus in various pathological conditions [13–16]. The anterolateral approach allows supine positioning, which is the most notable advantage for patients with multiple injuries [16]. According to Orthopaedic Trauma Association (OTA) techniques, the anterolateral approach is frequently used for lateral plating, which includes the risk of secondary nerve injury [17, 18]. Anterior plating was delineated years later (Fig. 2a-d) [19]. However, there are limited reports on the use of an anterolateral approach for the surgical treatment of midshaft fractures with anterior plating. Therefore, we conducted this study to assess the results of the treatment of a series of our patients with humeral midshaft fractures with anterior plate fixation and compare these results to those achieved in patients treated with posterior plating regarding the healing rate and occurrence of procedure-related complications. We hypothesized that compared to posterior plating, the anterolateral approach with anterior plating results in an equal union rate, a reduced rate of secondary nerve palsy and an equal rate of primary nerve palsy remission.

## Methods

**Fig. 3:**
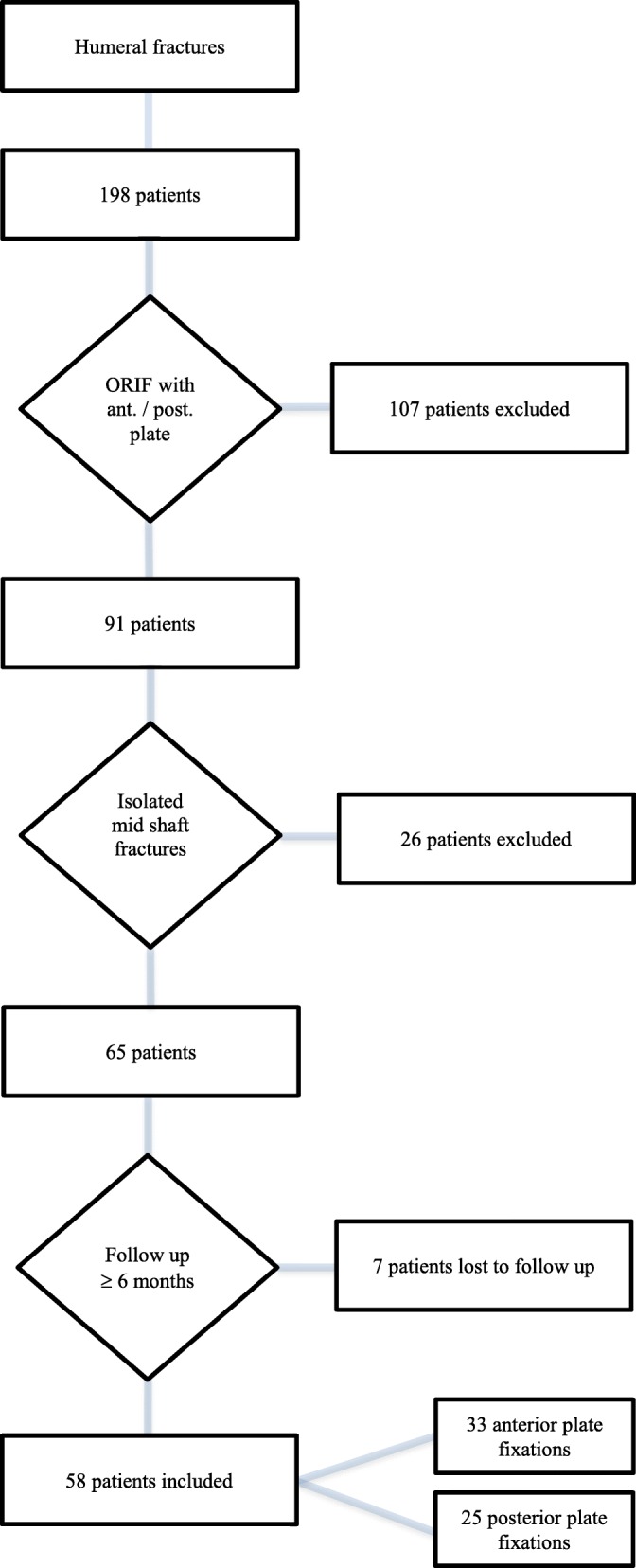
Inclusion and exclusion criteria

Approval for this study was granted by the local ethics committee (Reg. Nr. 16–5617-BR). The local electronic medical database was searched for patients 18 years of age or older with humeral midshaft fractures who underwent ORIF with anterior or posterior plate fixation at our institution. The inclusion and exclusion criteria are listed in Figure 3. The indication for the use of an anterior or posterior approach was based on the fracture pattern and concomitant soft tissue injuries according to the surgeons’ judgment. Further clinical data, including the demographics of each patient, were gathered. All fractures were classified according to the AO / OTA classification system [20]. For open fractures, the Gustilo / Anderson classification system was employed [21].

### Follow-up and radiographic evaluations

Clinical and radiographic follow-up examinations were performed six and twelve weeks after surgery. Subsequent additional visits were scheduled at different intervals until bone union was radiographically confirmed. The radiological results were assessed using anteroposterior and lateral radiographs. The radiographs were reviewed independently by two fellowship-trained orthopedic trauma surgeons and one radiologist. The primary outcome measure was bony healing after ORIF. Union was defined as a radiologically detectable callus bridge or at least three visible cortices on the radiographs. Depending on the type of callus formation, fracture healing was denoted as direct fracture healing (absolute stability: no callus formation) or indirect fracture healing (relative stability: callus formation) [22]. Nonunion was defined as failed fracture healing six months after the initial trauma. It was assessed clinically by the presence of pain and radiographically by the absence of a callus bridge or the persistence of visible fracture lines. The secondary outcome measures included the operative duration and rate of primary nerve palsy remission, secondary radial nerve palsy and infection. Posttraumatic nerve palsy was defined as primary nerve palsy, while postoperative palsy was defined as secondary nerve palsy. The neurological status was determined by a clinical examination performed by a neurological physician; in cases of radial nerve palsy, an electrophysiological assessment (ENG) was performed. We defined the complete absence of brachioradialis contraction with wrist drop in addition to a pathological ENG result without any potential as complete palsy. Palsy with pathologically reduced but detectable potentials and contractions of the brachioradialis was defined as incomplete palsy. Infection was defined by positive clinical signs of an infection, such as local pain, erythema, warmth, swelling and draining wounds in the affected limb leading to revision surgery [23].

### Statistical analysis

Patient characteristics are described by the mean, standard deviation and minimum and maximum values. The normality of variables was tested with the Shapiro-Wilk test. Significance was calculated using the t-test, Wilcoxon-Mann-Whitney test, contingency tables and Fischer’s exact test; *p* values of 0.05 or less were considered statistically significant. Data were analyzed using SPSS version 23 and Microsoft Excel version 16.22.

### Surgical technique

Trauma fellowship-trained orthopedic surgeons (trainee registrars or junior consultants) performed the surgeries in both groups.

**Fig. 4:**
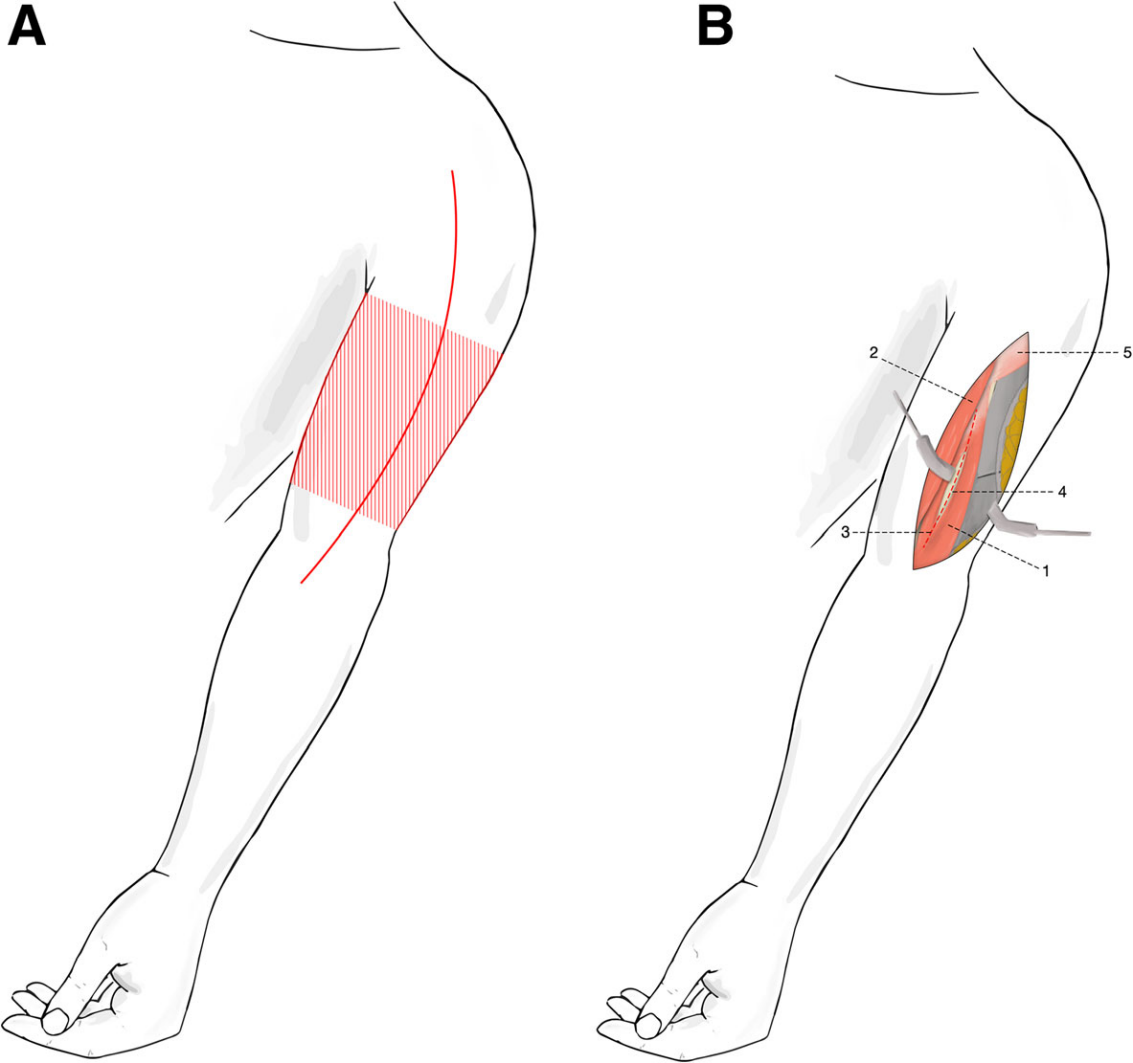
**a–b.** For exposure of the midshaft, only a portion of the approach is needed following the line of the lateral border of the biceps muscle (hatched area) (a). The biceps muscle is retracted medially, and the anterior aspect of the brachialis muscle is exposed. Dissection of the *M. brachialis* is performed to expose the bone (dashed line) (b). 1 Brachial muscle; 2 Biceps muscle of the arm; 3 Dissection of brachial muscle (dashed line); 4 Humerus; 5 Deltoid muscle

The approach was performed with the patient in the supine position on the operating table and the injured arm draped freely on an arm board. The landmarks for the skin incision in the anterolateral approach were the coracoid process of the scapula and the lateral boarder of the biceps muscle. For exposure of the midshaft, only a portion of the approach was needed following the line of the lateral border of the biceps muscle (Fig. 4a). A straight skin incision at the lateral border of the biceps muscle was made. Upon making an incision in the deep fascia of the arm in line with the skin incision, the muscular interval between the biceps brachii and brachialis muscles was identified. The biceps muscle was retracted medially, and the anterior aspect of the brachialis muscle was exposed. Dissection of the *M. brachialis* was performed to expose the bone (Fig. 4b). The radial nerve was visualized through the fracture gap to preclude nerve damage at the fracture site. Further exposure of the radial nerve was not performed.

In the posterior approach, the skin incision followed a line from the olecranon to the proximal third of the posterior arm. The fascia was divided along the same line. The lateral and long heads of the triceps were identified, and deep dissection was performed via splitting of the triceps muscle. The radial nerve was identified with its accompanying vessels piercing through the lateral intermuscular septum and followed proximally to where it crosses the humerus in its intermediate third. Distally, the common triceps tendon was split to expose the distal third of the posterior humeral shaft.

## Results

We included 58 patients with 58 fractures; the mean patient age was 59.9 years (range, 19–97 years). The mean follow-up duration was 34.6 months (range, 6–103) with a median of 28 months. In 33 cases (56.9%), anterior plating was performed, whereas in 25 cases (43.1%), plating was performed with a posterior approach. Further clinical data for both groups are shown in Table 1.

**Table 1:** Clinical data

Plate location:	anterior (Group A)	posterior (Group B)
Number of patients	N = 33	N = 25
Age	64.2 ± 19.5	54.8 ± 25.2
BMI	26.7 ± 5	27.61 ± 5.6
ASA	2.2 ± 0.7	2 ± 1
Sex		
male	16	14
female	17	11
OTA – classification		
Type A	22 (67.65%)	16 (64%)
Type A1	11	12
Type A2	–	2
Type A3	11	2
Type B	7 (20.59%)	7 (28%)
Type B1	3	5
Type B2	2	2
Type B3	2	2
Type C	4 (11.76%)	2 (8%)
Type C1	2	2
Type C2	2	–
Type C3	–	–
Open fracture	4 (12.12%)	2 (8%)
Gustilo type 1	4	1
Gustilo type 2	–	–
Gustilo type 3	–	1
Primary radial palsy	5 / 33 (15.15%)	7 / 25 (28%)

Union was achieved in 57 of 58 patients (98%). In total, 32 of 33 fractures healed after anterior plating (96.97%). Due to atrophic pseudarthrosis, one revision surgery was performed. In this case (1° open fracture), initial temporary external fixation was performed followed by anterior plating after wound closure. Union was achieved after revision with autologous iliac cancellous bone grafting. All 25 fractures (100%) healed after posterior plating. No significant difference was found in the union rate between the groups (*p* = 0.4). In 50 cases, healing was defined as secondary healing with callus formation. In seven cases, healing was defined as primary fracture healing. The mean operative duration for anterior plating was 96 min (range, 58–180). The mean operative duration for dorsal plating was 114 min (range, 56–238). No significant difference was found in the operative duration between the two groups (*p* = 0.19). Twelve of the 58 fractures (20.7%) were associated with primary radial nerve palsy prior to the index procedure. In all twelve cases, the palsy was defined as incomplete palsy with preserved but pathological ENG potentials. In the cases of nerve palsy, both the anterior (*n* = 5) and dorsal (*n* = 7) approaches had been used. Complete disruption of the radial nerve on surgical exploration (neurotmesis) was not found in any of these twelve cases. In ten of the twelve cases (83.3%), the palsy recovered completely after the index surgery. Recovery was achieved in four of five cases (80%) after an anterior approach had been used and six of seven cases (85.7%) after a dorsal approach had been used. No significant difference was found in the rate of primary nerve palsy remission between the two groups (*p* = 0.6). A total of four patients developed secondary incomplete radial nerve palsy following surgical treatment (8.69%); one out of 28 patients after anterior plating (3.57%) and three out of 18 patients after posterior plating (16.67%). No significant difference in the prevalence of secondary nerve palsy was found between the two clinical groups (*p* = 0.4). One patient showed no signs of remission after posterior plating, resulting in persistent complete palsy with wrist drop. Contrary to recommendations, the patient did not agree to undergo further surgical treatment. In three patients, full recovery was achieved spontaneously within six months. According to the noted complications, no infections were detected in either group (*p* = 1).

## Discussion

The purpose of this study was to evaluate a series of patients with humeral midshaft fractures treated with internal plate fixation regarding the effect of the approach and plate location (anterior vs. posterior) on the rate of healing, primary nerve palsy remission, iatrogenic nerve palsy and postoperative complications. Both groups showed a high healing rate with limited postoperative complications. According to the assessed outcome parameters, no significant differences were found between the two clinical groups.

In two recent reviews, Clement and Gosler et al. demonstrated a deficiency in the current literature of level one evidence for the treatment of humeral shaft fractures [3, 5]. Papasoulis et al. reviewed the available literature in 2010 and stated that the union rate ranged from 77 to 100% and good functional results were achieved after the nonsurgical treatment of humeral shaft fractures [24–26]. Nevertheless, a recent prospective randomized trial, published by Matsunaga et al. in 2017, provided level one evidence comparing functional bracing and bridge plating for humeral shaft fractures and showed that nonsurgical treatment was associated with a significantly higher rate of nonunion and angular displacement (anteroposterior) than bridge plating [8]. According to the current literature, there is no strong evidence to support the use of ORIF or minimally invasive procedures (MIPO) for primary fracture treatment. Xuqi Hu et al. presented the results of a systematic review and meta-analysis of eight studies, including four randomized controlled trials (RCTs), two prospective cohort trials and two retrospective cohort trials [27]. Of these eight studies, four compared ORIF to MIPO, and none of the four studies showed a significant difference in terms of the postoperative radial injury incidence, union rate or functional outcome between the two groups [28–31]. We have been using the anterior humeral approach frequently for the treatment of humeral shaft fractures by ORIF as well as for nonunion repair [14]. ORIF offers the opportunity for the exact reduction and anatomical fixation of the fracture and can enable primary or secondary fracture healing depending on the type of osteosynthesis and fracture pattern. Although there were no significant differences between our two groups according to the primary and secondary outcome measures, an anterior approach offers advantages. It allows supine positioning of the patient and offers safe exposure of the humerus as the radial nerve is not directly explored [10]. To the best of our knowledge, there have been no prospective randomized studies comparing anterior and posterior plate fixation in terms of the healing rate and clinical outcomes. Nevertheless, the currently available literature confirms our finding that an anterior surgical approach with plating is a safe and efficacious treatment option for humeral shaft fractures. Reliable results have been reported in one biomechanical study [32] and one retrospective clinical study [33] for anteromedial plating for shaft fractures in the upper extremities with regard to bone union and iatrogenic neurovascular injury. One retrospective study of 96 humeral fractures treated with anteromedial plating presented a union rate of 97%, although 20% of the fractures included were open fractures [16]. According to the neurological status, 18 patients with primary radialis palsy and one patient with brachial plexopathy were included in this study. Of these 19 patients, twelve achieved remission after ORIF. Two patients (2.1%) were noted to have secondary palsy (hypoesthesia in the lateral antebrachial cutaneous nerve distribution) after surgery. Another retrospective study was published by Boschi et al. [15] investigating the outcomes of the treatment of 280 humeral shaft fractures with ORIF in terms of the approach and plate location. The overall healing rate was 98.2%, without a significant difference in the approach or plate location. In accordance with the findings reported by Boschi et al. [15], no significant difference in the operative duration was found between the two groups in our study; however, we found a wide variation in the operative duration within the groups. As a level one trauma center and a university hospital, all operative procedures in both groups were performed by either trainee registrars or junior consultants, which might be one reason for the wide variation in the operative duration within the groups. The fact that the number of surgeons and their level of experience did not affect the outcome with respect to healing and complications underlines the safety of the procedure and the reproducibility of the results rather than representing a limitation in terms of interpreting the results of the study.

Humeral shaft fractures are commonly associated with lesions of the radial nerve. The anatomical proximity and association of the bone and nerves in the humeral shaft explain the incidence of between eight and 12 % [34, 35]. We documented primary radial nerve palsy in twelve of the 58 patients (20.7%). The best treatment for humeral shaft fractures complicated with radial nerve injury is highly controversial [36, 37]. While concomitant nerve injury has been used as an argument for the immediate surgical treatment of fractures in the past (using a posterior approach and visualizing the radial nerve) [38], recent investigations have shown no significant difference in radial nerve palsy recovery between initial operative and nonoperative management strategies [34, 39]. In accordance with these findings, we found no significant difference in the remission rate between the use of a posterior approach in conjunction with revealing the radial nerve and the use of an anterior approach without nerve exploration. Most radial nerve injuries in cases of humeral shaft fracture are caused by traction or compression of the nerve, which is known as neuropraxia. Much fewer nerve injuries are identified as discontinuity of the nerve (axonotmesis or neurotmesis) [40]. Neuropraxia is a reversible injury, resulting in spontaneous reversibility in a large portion of traumatic radial nerve palsy cases [34], which underlines our findings that even in cases of fracture with primary radial palsy, an anterior approach with plating is a feasible alternative to a posterior approach. However, certain studies have described significant soft tissue damage related to the use of an anterior approach. Cutting through the brachialis muscle may lead to the loss of muscle strength and the loss of tension on elbow flexion [15, 41]. Additionally, the danger of iatrogenic damage to the radial nerve (innervating the lateral aspect of the muscle during distal dissection) and musculocutaneous nerve (entering the superior third of the brachialis muscle and innervating the medial aspect) has been described [42–44]. However, we did not detect any adverse effects on the musculocutaneous nerve resulting from anterior plating in this study. Although we detected a lower rate of secondary nerve palsy in the anterior plating group than in the posterior plating group (3.57% vs. 16.67%), this difference was not significant. We acknowledge that there have been contradictory descriptions of the incidence of postoperative radialis palsy with the use of an anterior approach in recent studies, reportedly ranging from 11 to 16% [15, 45]. Gouse et al. [45] reported a study including 37 closed humeral shaft fractures and 29 humeral fractures with nonunion after plating fixation with an anterolateral approach. In total, eleven of the 66 patients (16%) developed secondary radial nerve palsy. Gouse et al. [45] stated significance according to risk factors for suffering nerve palsy based on the surgical experience of the surgeon and timing of the surgery.

The main limitations of the current study are based on its retrospective design without a defined follow-up protocol and an evaluation of the operating surgeons with varying levels of experience. Although no long-term complications occurred in this study and the primary and secondary outcome measures were addressed sufficiently*,* one limitation is the minimum follow-up time by six months. Second, the patients were observed at individual intervals after the twelve-week visit, so postoperative data, such as healing time, could not be gathered without bias. Third, even though the descriptive statistics suggest that anterior plating reduces the risk of secondary nerve palsy compared to posterior plating (1 / 28; 3.57% vs. 3 / 18; 16.67%), the number of included patients might have been too low to show a significant difference.

## Conclusions

ORIF using an anterior approach with plate fixation provides a safe alternative to posterior plating in the treatment of humeral shaft fractures. An anterior approach allows supine positioning of the patient and yields comparable union and complication rates compared to a posterior approach with plate fixation for treating humeral shaft fractures.

## Data Availability

The datasets of the current study are not publicly available. Data are available upon request from the first author, SL.

## Abbreviations

ENG: Electrophysiological assessment
LCDCP: Limited contact dynamic compression plate
MIPO: Minimally invasive plate osteosynthesis
ORIF: Open reduction internal fixation
OTA: Orthopaedic Trauma Association
RCT: Randomized controlled trial
